# Modulation of Respiratory System by Limb Muscle Afferents in Intact and Injured Spinal Cord

**DOI:** 10.3389/fnins.2019.00289

**Published:** 2019-03-26

**Authors:** Natalia A. Shevtsova, Vitaliy Marchenko, Tatiana Bezdudnaya

**Affiliations:** Department of Neurobiology and Anatomy, Drexel University College of Medicine, Philadelphia, PA, United States

**Keywords:** respiration, muscle stimulation, exercise, muscle afferents, spinal cord injury

## Abstract

Breathing constantly adapts to environmental, metabolic or behavioral changes by responding to different sensory information, including afferent feedback from muscles. Importantly, not just respiratory muscle feedback influences respiratory activity. Afferent sensory information from rhythmically moving limbs has also been shown to play an essential role in the breathing. The present review will discuss the neuronal mechanisms of respiratory modulation by activation of peripheral muscles that usually occurs during locomotion or exercise. An understanding of these mechanisms and finding the most effective approaches to regulate respiratory motor output by stimulation of limb muscles could be extremely beneficial for people with respiratory dysfunctions. Specific attention in the present review is given to the muscle stimulation to treat respiratory deficits following cervical spinal cord injury.

## Limb Muscle Afferents and Respiration

Experimental studies of respiratory modulation via peripheral muscles can be divided into two overlapping groups: (i) investigation of functional interactions between locomotor and respiratory neural circuits and (ii) studying the effect of muscle afferent activation on cardio-respiratory system during exercise.

### Functional Interaction Between Locomotion and Respiration

**Figure 1:**
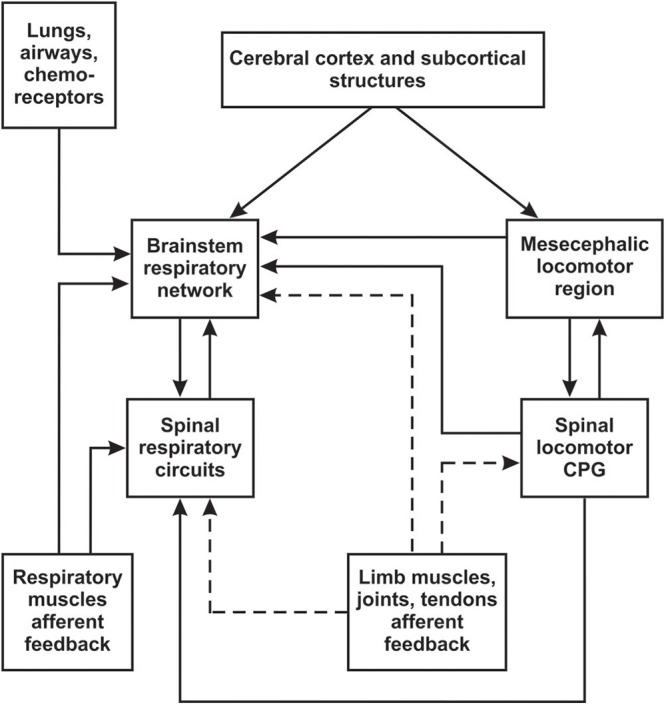
Schematic diagram of possible interactions between locomotor and respiratory systems. Brainstem respiratory network includes medulla and pons (see Section “Overview of Respiratory and Locomotor Networks”). Afferent inputs from limb muscles, joints and tendons are outlined by dashed line.

Many experimental studies have demonstrated that locomotion is functionally linked to respiration by coupling between central pattern generators (CPGs) for stepping and breathing (Boggs, 2002). This coupling has often been attributed to rhythmic activation of sensory inputs from the limbs during muscle contraction, affecting the activity of the respiratory CPG (Morin and Viala, 2002; Potts et al., 2005; Giraudin et al., 2008, 2012). It is important to note, however, that there are also central mechanisms of interaction between locomotor and respiratory systems. These include feedforward control of locomotion and respiration by cerebral cortex and subcortical structures (Krogh and Lindhard, 1913; Eldridge et al., 1981b, 1985; DiMarco et al., 1983; Waldrop et al., 1986), direct inputs to the respiratory CPG from the mesencephalic locomotor region (MLR) (Gariepy et al., 2012), interactions between spinal locomotor and brainstem respiratory CPGs (Persegol et al., 1988; Le Gal et al., 2014; Yazawa, 2014), or spinal locomotor and spinal respiratory circuits (Viala et al., 1979; Viala, 1986). The potential interactions between locomotor and respiratory systems are outlined in Figure 1. While the central mechanisms are significant, the focus of this review will be on studies of respiratory modulation by peripheral mechanisms (limb muscle afferents), with consideration to how these can be therapeutically targeted.

In the experimental setup to study afferent inputs from limb muscles to respiratory system, activation of muscle afferents can be achieved by nerve, muscle or dorsal roots electrical stimulation as well as by passive or active limb movements or stretch (Koizumi et al., 1961; Palisses et al., 1988; Persegol et al., 1988; Potts et al., 2005; Giraudin et al., 2008; Le Gal et al., 2016).

During experimental stimulations of limb muscle afferents, two major effects on respiration can be observed: entrainment of breathing and hyperpnoea. During entrained breathing the respiratory rhythm resets and follows the frequency of stimulus applied to muscle afferents (Iscoe and Polosa, 1976; Iscoe, 1981; Morin and Viala, 2002; Potts et al., 2005), whereas hyperpnoea is characterized by increased frequency of breathing and tidal volume (Koizumi et al., 1961; Tibes, 1977; Persegol et al., 1993). Entrainment of breathing is more related to locomotor-respiratory coupling, while hyperpnoea to exercise in general. Both supraspinal and spinal mechanisms could be involved in these respiratory responses.

### Overview of Respiratory and Locomotor Networks

The supraspinal respiratory network is organized into several brainstem compartments that extend from the lower medulla to the pons (for review see Rybak and Smith, 2009; Smith et al., 2009). Respiratory rhythm originates within the ventral respiratory column (VRC) in the medulla. The VRC includes the Bötzinger Complex (BötC), pre-Bötzinger Complex (pre-BötC), rostral (rVRG) and caudal (cVRG) subregions of the ventral respiratory group (VRG). The pre-BötC contains mostly inspiratory neurons and is considered a primary source of rhythmic inspiratory activity. The BötC, containing mostly post-inspiratory and expiratory neurons, together with the adjacent pre-BötC represent a kernel of the respiratory CPG that is critically important for the generation of the basic respiratory activity. The pre-BötC and BötC receive multiple drives from other medullary and pontine regions, such as the parabrachial and Kolliker-Fuse nuclei (PBN/KF), comprising the pontine respiratory group (PRG), the retrotrapezoid nucleus/parafacial respiratory group (RTN/pFRG), raphe nucleus, nucleus tractus solitarius (NTS) in medulla and others. The NTS and adjacent reticular neurons form the dorsal respiratory group (DRG). The emerging view is that the VRC, DRG, and PRG collectively constitute the brainstem respiratory network. The activity of brainstem respiratory network is modulated by feedback from neurons in other regions of the brain, brainstem, spinal cord and periphery. The NTS processes peripheral chemo- and lung stretch receptors afferent feedback and provides increase in breathing (chemoreflex) or decrease in amplitude/duration of inspiration (Hering–Breuer reflex), respectively. RTN/pFRG is important for central chemoreception and formation of active expiratory pattern. The PBN/KF nuclei regulate the inspiratory-expiratory phase transition and mediate somatic afferent feedback from peripheral (limb) muscles to the VRC compartments of the respiratory CPG.

The primary spinal respiratory circuits comprise phrenic, intercostal, and abdominal interneurons and motoneurons, innervating the associated primary respiratory muscles (see Lane, 2011). These circuits receive inputs from the brainstem respiratory center and segmental peripheral afferent inputs (Sumi, 1963; Smith et al., 2009). In particular, the pre-BötC projects to the bulbospinal inspiratory neurons in the rVRG relaying the inspiratory drive to phrenic (PhMNs) and intercostal motoneurons innervating the diaphragm and external intercostal muscles. Bulbospinal expiratory neurons in the cVRG receive convergent inputs from the BötC and RTN/pFRG and shape expiratory drives to spinal thoracic and lumbar expiratory motoneurons. In addition, the spinal respiratory circuits receive the afferent feedback from respiratory muscles, and, as was shown experimentally, afferent inputs from limb muscles, either directly or via spinal locomotor circuits (Morin and Viala, 2002; Giraudin et al., 2012; Le Gal et al., 2014).

The spinal locomotor circuits represent a bilateral neural network located in the cervical and lumbar spinal segments, which are capable of generating the basic locomotor rhythm and shaping locomotor activity (Falgairolle et al., 2006; Kiehn, 2006; Guertin, 2012). This network includes the rhythm generating neurons, spinal interneurons and motoneurons, controlling activity of limb muscles. The spinal locomotor circuits receive sensory feedback from limb muscles afferents, inputs from the brainstem MLR and from higher brain structures. The activity of the left and right cervical and lumbar CPGs is coordinated by commissural and long propriospinal interneurons. In addition to these interneuronal populations, spinal projection neurons innervate supraspinal regions, relaying sensory afferent input from limb muscles.

### Limb Muscle Afferent Inputs to the Supraspinal Respiratory Network

Involvement of the supraspinal networks in locomotor-respiratory interaction suggests that sensory feedback from limb muscles can reset, entrain or facilitate the brainstem respiratory CPG (Morin and Viala, 2002; Potts et al., 2005; Giraudin et al., 2012). While supraspinal mechanisms of exercise hyperpnoea will be discussed in more details below, here we will focus on the entrainment of breathing. Repetitive stimulation of muscle afferents can produce 1:1 entrainment of the respiratory frequency (Kawahara et al., 1988; Palisses et al., 1988; Morin and Viala, 2002; Potts et al., 2005). Animal experiments have shown that the entrainment of respiratory rhythm is phase-dependent and restricted to stimulation during the expiratory phase (Kawahara et al., 1988; Potts et al., 2005). Studies in brainstem-spinal cord preparation of newborn rats *in vitro* have demonstrated that afferent inputs from the hindlimb muscles can be transmitted to spinal locomotor CPG and then to the brainstem (Le Gal et al., 2014) or directly to the brainstem respiratory network bypassing the lumbar locomotor CPG (Morin and Viala, 2002; Giraudin et al., 2012). This suggests that activation of the spinal locomotor CPG is not required for respiratory rhythm entrainment during rhythmic stimulation of limb afferents (Morin and Viala, 2002). Experiments with reverse blockade of the PBN (Potts et al., 2005) and brainstem transections (Giraudin et al., 2012) showed an inability to entrain breathing, implicating the pivotal role of PBN neurons in the locomotor-respiratory coupling during afferent stimulation of limb muscles when spinal locomotor CPG is not activated. Anatomical studies in rats confirmed the existence of afferent inputs to the PBN from the dorsal spinal cord and the spinal trigeminal nucleus (pars caudalis), mainly from lamina I (Cechetto et al., 1985; Bernard et al., 1995; Craig, 1995). Immunohistochemical and electrophysiological studies have shown that most PBN neurons receiving these inputs respond to nociceptive stimulation (Standaert et al., 1986; Bernard et al., 1994; Hermanson and Blomqvist, 1996). It was suggested that the PBN receives information from somatic nociceptive receptors and adjusts cardio-respiratory responses to their activation (Gauriau and Bernard, 2002). Thus, it is unclear if observed respiratory rhythm entrainment via the PBN is a specific result of stimulation of nociceptive afferents or it can be generally implied to activation of non-nociceptive muscle afferents. Experiments to selectively block different afferent subtypes are needed to confirm that nociception is not involved in respiratory entrainment. This raises an important question: what type of skeletal muscle afferents should be activated to trigger respiratory responses?

### Muscle Afferent Fiber Types

Muscle afferents are represented by axons of sensory neurons that reside within the spinal dorsal root ganglion. These pseudo-unipolar neurons transfer sensory information from muscles to the spinal cord via spinal nerves. According to Lloyd’s classification (Lloyd, 1943), there are four major types of muscle afferent fibers [I (Ia and Ib), II, III, and IV], differing by their physiological characteristics as well as by the sensory inputs activating them. Specifically, type Ia fibers, located in the muscle spindles, respond to activation of rapidly adapting stretch receptors and signal the fast change in muscle length and velocity. Type Ib fibers respond to activation of Golgi tendon organ and signal the change in muscle force tension. The type I (Ia and Ib) fibers are thickly myelinated (12–20 μm), have a large diameter and fast conductive velocity (70–120 m/s in cats and dogs) (Lloyd, 1943; Whitwam, 1976; McCord and Kaufman, 2010). Type II fibers respond to activation of slow non-adapting stretch receptors and provide information about muscle length and limb position. They are also thickly myelinated (6–12 μm), but have slower conductive velocity than type I (30–70 m/s). Type III and IV free ending fibers provide sensory feedback related to different physiological conditions of a muscle: mechanical contractive and metabolic (byproducts of muscular work, e.g., potassium, prostoglandins, lactic and arachidonic acids, ATP et al.) conditions, including noxious sensation (McCord and Kaufman, 2010; Mense, 2010; Kaufman and Forster, 2011; Forster et al., 2012; Pedersen, 2013). Type III fibers are represented by thinly myelinated, small diameter (1–6 μm) and lower conductive velocity axons (2.5–30.0 m/s) and type IV – by unmyelinated, smallest diameter (less than 1 μm), lowest conductive velocity fibers (<2.5 m/s). Sensory responses to mechanical stimulation are provided mostly by type III fibers, whereas metabolic changes during muscular work are sensed by type IV fibers (Kaufman et al., 1983, 1984; Hayes and Kaufman, 2001). It has been demonstrated that the type III/IV non-nociceptive muscle fibers can be activated by both static and dynamic muscle contractions and respond to all types of movements including exercise (Kaufman et al., 1983, 1984).

Experimental studies suggest that entrainment of respiratory rhythm and exercise hyperpnoea could be triggered by different afferents. Studies in the newborn isolated brainstem-spinal cord preparation (Morin and Viala, 2002; Giraudin et al., 2008, 2012) have demonstrated that type I/II afferent fibers are responsible for respiratory rhythm entrainment. Here, we should keep in mind that results in newborn preparations *in vitro* might be different from adult motor circuits *in vivo* (Smith and Feldman, 1987). However, the major role of type I-II afferents in entrainment was supported by some *in vivo* investigations (Koizumi et al., 1961), but not all (Iscoe and Polosa, 1976; Kawahara et al., 1988). In addition, both experimental and clinical studies have revealed that stimulation of primary afferents (including type I-II) can entrain the spinal locomotor CPG (Kiehn et al., 1992; Guertin et al., 1995; Dietz et al., 2002; Lev-Tov et al., 2010) and, thus, may elicit ventilatory changes via direct interaction between the spinal locomotor and brainstem respiratory CPGs (Persegol et al., 1988; Le Gal et al., 2014; Yazawa, 2014), or spinal locomotor and spinal respiratory circuits (Viala et al., 1979; Viala, 1986) (see Figure 1). In contrast, however, most investigators believe that exercise hyperpnoea is triggered exclusively by stimulation of type III/IV fibers (Mateika and Duffin, 1995; Amann et al., 2010; Kaufman, 2010; Amann, 2012). Experiments with selective blocking and deactivating of the different afferent types have shown that activation of type III/IV non-nociceptive fibers significantly contribute to changes in ventilation during limb muscle activity (McCloskey and Mitchell, 1972b; Tibes, 1977; Amann et al., 2010).

### Limb Muscle Afferent Inputs to the Spinal Respiratory Network

Though neural mechanisms responsible for generation of the respiratory rhythm and pattern have been extensively studied for many years, most of these studies were focused on respiratory circuits in the brainstem (Feldman et al., 2003; Smith et al., 2009, 2013). Less attention has been given to spinal neural circuits involved in modulation of the respiratory activity. Accordingly, their organization and functional contribution are poorly understood. Nevertheless, the importance of spinal interneurons for regulation of respiration in the intact and injured spinal cord has been demonstrated (Sumi, 1963; Palisses et al., 1989; Bellingham and Lipski, 1990; Lane et al., 2008, 2009; Lois et al., 2009; Sandhu et al., 2009; Marchenko et al., 2015; Zholudeva et al., 2017). Specifically, a subset of spinal interneurons (distributed mostly in V-VII and X layers of Rexed) that modulate the activity of phrenic motoneurons (PhMNs), controlling the contraction of the diaphragm – the main muscle of respiration, has been identified (Yates et al., 1999; Lane et al., 2008, 2009; Lois et al., 2009). Overall, respiratory spinal interneurons have been found to be involved in many functions including intra-/intersegmental and left-right coordination of respiratory motor outputs, integration of different motor activities, and promotion of respiratory plasticity following cervical spinal cord injury (Sumi, 1963; Palisses et al., 1989; Bellingham and Lipski, 1990; Lane et al., 2008, 2009; Sandhu et al., 2009; Darlot et al., 2012; Zholudeva et al., 2017). The diversity of spinal interneuron phenotypes and their known contributions to breathing are highlighted in Zholudeva et al. (2018).

**Figure 2:**
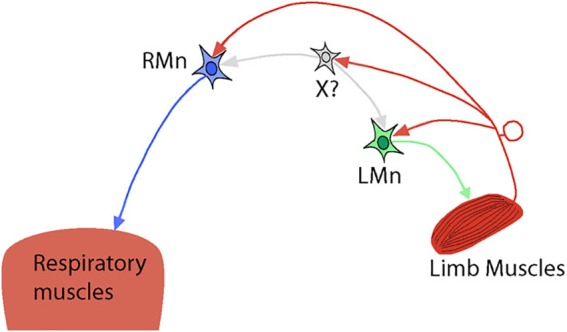
Simplified hypothetical diagram representing intraspinal connections between locomotor and respiratory spinal circuits. RMn – respiratory motoneuron. LMn – limb motoneuron. X? – shared interneuron that drives/modulates both RMn and LMn and receives afferent feedback from limb muscles. Possible direct inputs to RMn from limb muscle afferents are also shown.

However, the spinal mechanisms of interactions between locomotion and respiration are not well studied. It is believed that the spinal mechanisms can modulate ventilation via specific connections between afferent inputs from limb muscles and respiratory neural circuits at the spinal cord level (Koizumi et al., 1961; Eldridge et al., 1981a; Morin and Viala, 2002; Le Gal et al., 2016). The experimental results allow to suggest the existence of “shared” spinal interneurons that receive afferent feedback from limb muscles and are involved in both locomotor and respiratory spinal circuits (see Figure 2). Indeed, experiments in the isolated brainstem-spinal cord of newborn rats have shown excitatory connections between the lumbar locomotor CPG, which receives afferent feedback from muscles, and expiratory motor- and interneurons located at the lumbar and thoracic levels in the spinal cord (Le Gal et al., 2016). The activity of these neurons can be rhythmically modulated by the locomotor activity induced either pharmacologically or by afferent stimulation (Le Gal et al., 2016). These connections are exclusively intraspinal and, as authors suggest, may assist in the coordination of locomotion and respiration by facilitation of expiratory efforts.

Another study performed on the same brainstem-spinal cord preparation with intracellular recordings of PhMNs has demonstrated that ascending afferent fibers from hindlimb muscles give collaterals to these motoneurons (Morin and Viala, 2002). Activation of hindlimb afferents results in a monosynaptic short-latency activation of PhMNs, followed by their disynaptic inhibition via GABA-ergic spinal interneurons and polysynaptic excitation of a supraspinal origin (through the spinal-bulbar-spinal loop). To explain the disynaptic inhibitory effect on PhMN activity, the authors propose that inputs from limb afferents are necessary to “prepare the PhMN population to receive excitatory polysynaptic commands via the medullary respiratory network” (see Figure 7 in Morin and Viala, 2002). The role of the monosynaptic activation of PhMNs by afferent stimulation was not discussed in this study and could be addressed in future research by pharmacological disinhibition of PhMNs in a spinal preparation and rhythmic stimulation of limb muscles to see if this can elicit phrenic nerve activity and diaphragm contractions. However, one experimental work in decorticate and spinal rabbits was done in this direction and has shown that following activation of spinal cord with DOPA, periodic hindlimb movements were able to elicit large bursts in phrenic nerves, supporting the idea about direct excitatory inputs from hindlimb muscles afferents to PhMNs (Palisses et al., 1988) and their inhibition in intact spinal cord.

*In vivo* experiments in spinal cats (without supraspinal control) have demonstrated inhibitory effect of hindlimb afferent stimulation on phrenic (Eldridge et al., 1981a) and intercostal motoneuron activity (Koizumi et al., 1961). In the study by Eldridge et al. (1981a), the authors used intraspinal intercostal-to-phrenic reflex to evoke phrenic bursts in C1 transected cats by electrical stimulation of intercostal nerves or rhythmic manual compression/tapping of the lower thorax described by Decima et al. (1969). Continuous physical stimulation of the calf muscle or electrical stimulation of the tibial nerve for 60 sec produced inhibition of the evoked phrenic activity. The authors did not evaluate the type of afferents activated by this stimulation but suggested that small myelinated and unmyelinated fibers were involved. Interestingly, the extent of inhibition declined over the course of stimulation. Moreover, a brief increase in phrenic activity (rebound) was observed after stimulation. The decline in inhibition and rebound were attributed to accommodation and increase in neuronal excitability post-inhibition, which thought to be the common membrane properties of spinal motoneurons (Coombs et al., 1959; Sears, 1964). It should be noted that all studies considered above were focused on hindlimb afferent stimulation. Interactions between forelimb afferents and PhMNs have not been demonstrated yet.

Overall, these experiments confirm the presence of intraspinal connections between locomotor and respiratory motor systems. Interactions between supraspinal and spinal mechanisms of respiratory modulation by muscle afferent stimulation are not clear, however, and more experimental work is required.

### Cardio-Respiratory Control During Exercise

Mechanisms of cardio-respiratory responses during exercise have been extensively studied for over a hundred years (see review by Mitchell, 2013). Exercise increases the oxygen demands, production of carbon dioxide and different metabolites in working muscles. Elevated ventilation, heart rate, and blood pressure are required to match metabolic changes in the organism and are correlated with the intensity of exercise (D’Angelo and Torelli, 1971; Casey et al., 1987; Iellamo et al., 1999). Many studies refer to these changes as the “exercise pressure reflex” (Mitchell et al., 1983; Kaufman and Hayes, 2002). Several mechanisms have been suggested to explain the cardio-respiratory responses during exercise: (i) feedforward central commands (Eldridge et al., 1981b, 1985; Waldrop et al., 1986); (ii) peripheral neural regulation through type III and IV muscle afferents (McCloskey and Mitchell, 1972a) and (iii) central integration of inputs from the peripheral and/or central chemoreceptors (see review Gariepy et al., 2010). Experiments in human subjects have confirmed the significant modulatory role of type III/IV muscle afferents in cardio-respiratory responses during exercise by using the anesthetic (lidocaine, fentanyl) injections into the lumbar epidural space to reduce sensation from those afferents and observing the attenuation of cardio-respiratory responses during exercise or electrical stimulation of hindlimb (Strange et al., 1993; Kjaer et al., 1994; Amann et al., 2010; Dempsey et al., 2014). However, the debate on the role of type III/IV muscle afferents in cardio-respiratory responses during exercise continues (Poon and Song, 2015).

Three phases of ventilatory response to mild-to-moderate exercise have been distinguished in a healthy subject: (1) a rapid increase in total ventilation at the exercise onset; (2) a transient phase with a further continuous increase of ventilation; and (3) the final steady phase characterized by plateaued ventilation (Whipp et al., 1982; Turner, 1991; Bruce, 2017). The rapid increase in ventilation in phase 1 cannot be explained by changes in metabolic level. It was proposed that at the exercise onset, neuronal inputs from motor cortex (central feedforward mechanism) and/or stimulation of muscle/joint afferents lead to a fast increase of the respiratory CPG output resulting in hyperpnoea. As exercise continues, the humoral mechanism was suggested to perform further tuning of respiration (Dejours, 1964; Griffiths et al., 1986; Whipp, 1994). At exercise offset, the removal of central and afferent signals leads to abrupt fall in hyperventilation, followed by slow decay in ventilation until humoral changes are diminished. Control of respiratory parameters by the type III/IV afferents is likely to be involved in all phases of ventilatory response to exercise (Kaufman et al., 1983, 1984; Amann, 2012; Duffin, 2014).

**Figure 3:**
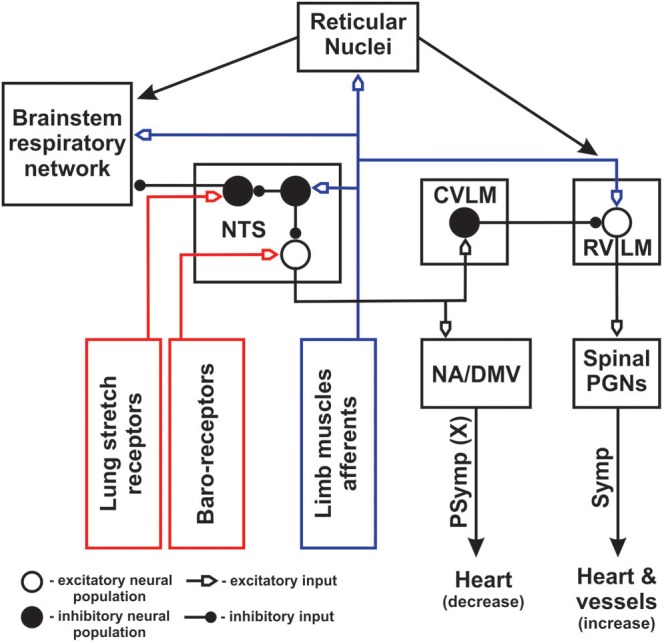
Schematic diagram of cardio-respiratory interactions during exercise. Blue color represents afferent feedback from limb muscles. Red color shows afferent feedback from baro- and lung stretch receptors. NA – nucleus ambiguous; DMV – dorsal motor nucleus of vagus; PGNs – preganglionic neurons; Symp – sympathetic outputs; PSymp – parasympathetic output; X – vagus nerve. Open and filled big circles represent excitatory and inhibitory neuron populations, respectively. Filled small circles show inhibitory inputs, open small arrows indicate excitatory inputs. NTS was moved out from the block presenting brainstem respiratory network to show its inputs and outputs in greater details. Peripheral/central chemoreception was not included for simplicity.

Anatomical and electrophysiological studies have shown that high threshold (III/IV type) muscle afferents have direct and non-direct projections (via spinal laminae I-V neurons) to the lower brainstem including the NTS (Kalia et al., 1981; Potts, 2001, 2006; Kawabe et al., 2007), reticular nuclei (Ciriello and Calaresu, 1977; Menetrey et al., 1983; Iwamoto et al., 1984) and rostroventrolateral medulla (RVLM) (Kawabe et al., 2007). It has been proposed that GABA-ergic neurons of the caudal NTS activated by somatic afferents may play a major role in inhibition of the baroreflex, which provides negative control on blood pressure and heart rate, resulting in increase in blood pressure and heart rate during exercise (Potts, 2001, 2006). We hypothesize that similar mechanism may inhibit inputs from lung stretch receptors, decreasing the strength of the Hering-Breuer reflex (by inhibiting of GABA-ergic pump cells projecting from the NTS to the respiratory CPG) (see Figure 3). On the one hand, direct activation of RVLM presympathetic neurons by muscle afferents during exercise can elicit an increase in blood pressure and heart rate. Suppression of the baro- and Hering-Breuer reflexes during exercise may lead to hypertension and increased tidal volume. On the other hand, it was demonstrated that activation of vagal afferents might inhibit the exercise effect in conscious rats (DiCarlo et al., 1994). Sensory feedback from baroreceptors and lung stretch receptors are mutually inhibitory with somatic afferents at the NTS, so the outcome of sensory stimuli will depend on the balance of all afferent inputs. Moreover, the recent work of Silva et al. (2018) has shown that simultaneous stimulation of muscle and peripheral chemosensory afferents during passive knee movement and hypoxia resulted in hyperadditive hyperventilation in healthy volunteers. These data may reflect the importance of integration between neurogenic (muscle afferents) and metabolic (peripheral chemoreceptors) inputs in the NTS and/or other structures of ponto-medullary respiratory network to provide an appropriate respiratory response to physical exercise.

In addition, polysynaptic ascending spinal pathways activated by somatic muscle afferents include the spino-reticular tracts. Spino-reticular neurons project to reticular structures in the vicinity of the ventrolateral medulla (Iwamoto et al., 1984, 1989; Iwamoto and Kaufman, 1987; Bauer et al., 1990) and can modulate the respiratory CPG (Jones et al., 2016), bulbospinal inspiratory/expiratory (Dobbins and Feldman, 1994) and presympathetic RVLM neurons (Ciriello and Calaresu, 1977; Iwamoto et al., 1989; Bauer et al., 1990; Babic and Ciriello, 2004; Guyenet and Stornetta, 2004) regulating cardiovascular function. Activation of these projections during exercise can directly change the frequency/amplitude of the breathing and increase blood pressure/heart rate. The conceptual schematic of the central interactions between somatic afferents and cardio-respiratory networks during exercise is shown in Figure 3.

### Effects of Different Muscles Stimulation

Stimulation of different muscles (hindlimb vs. forelimb or flexors vs. extensors) may have different effects on respiration during locomotion or exercise. Moreover, prior studies indicate that even activation of the same muscle can have inconsistent effects between animal species (Boggs, 2002). This is likely due to differences in the way these muscles are used in different species, for instance, the role of fore- and hindlimb muscles in bipedal and quadrupedal animals. In bipeds, hindlimbs carry 100% of static body weight whereas, in quadrupeds, forelimbs usually support about 55–60% of body weight during standing. Some quadrupedal animals can use their forelimbs to carry and manipulate objects, some do not. Worth noting, however, is that in most animals used in electrophysiological experiments (mice, rats, cats, rabbits, dogs), the hindlimb muscles have larger muscle mass than forelimbs. This fact makes hindlimb muscles more popular for experimental and clinical research (Koizumi et al., 1961; Iscoe and Polosa, 1976; Tibes, 1977; Viala, 1986; Viala et al., 1987; Palisses et al., 1988; Persegol et al., 1993). However, some investigations have used forelimb muscle stimulation as well (Potts et al., 2005). Nevertheless, electrical stimulation of dorsal roots at either cervical or lumbar levels in the isolated newborn rat brainstem-spinal cord preparation revealed a similar effect on respiration (Giraudin et al., 2008). The published results on flexor vs. extensor muscle afferent stimulation are contradictory. Specifically, some studies show a greater increase in respiratory output with hindlimb flexor stimulation (Persegol et al., 1993; Giraudin et al., 2012), while others report the opposite (Viala et al., 1987; Palisses et al., 1988), or even equal effects regardless of muscle stimulated (Koizumi et al., 1961). These contradictory results can be explained by using various experimental models and paradigms of muscle stimulation. Further studies are needed for clarification of the effect of different muscle stimulation on modulation of cardio-respiratory output.

Human studies suggest that the magnitude of cardiovascular and respiratory response during exercise might depend on the mass of a muscle involved (Iellamo et al., 1999). Therefore, in general, the effect on the ventilatory parameters is expected to be higher during lower limb vs. upper limb exercise. This could be explained by activation of a higher number of afferent fibers, more metabolites production and increased blood flow capacity in muscles with larger mass. However, many clinical studies have shown the same effect of upper vs. lower limb activation during exercises of the same intensity (Kang et al., 1997; Faria and Faria, 1998). One possible explanation of this inconsistency could be spatial distribution and activation of different muscle fiber types that were associated not only with different physiological but also metabolic properties (slow-twitch (type 1) fibers – resistant to fatigue vs. fast twitch (type 2) fibers that fatigue much faster, but produce more force). It was shown that the type 2 fibers (glycolytic) are more metabolically active than type 1 (oxidative) (Saltin and Gollnick, 1983; Casey et al., 1996). Therefore, stimulation of muscles with a higher proportion of type 2 fibers will likely produce a bigger increase in ventilation and cardiovascular response than stimulation of muscles with a higher proportion of type 1 fibers (see for review Fisher and White, 2004). In general, lower limb muscles in human have a higher proportion of slow-twitch fibers (type 1) compared to upper limbs (Johnson et al., 1973; Edgerton et al., 1975; Dahmane et al., 2005), perhaps, because of their role in bipedal locomotion. Thus, the effect on respiratory responses during upper limb vs. lower limbs exercising may be comparable given the opposing differences in the number of type 1–2 fibers and muscle mass, which could compensate each other.

Another consideration is that, even within lower limb muscles, there is a specific pattern of distribution of different type fibers. Deep extensor lower limb muscles predominantly contain type 1 fibers when more peripheral muscles have a higher proportion of type 2 fibers. This is correlated with the recruitment of muscles during locomotion: deep muscles are activated during postural standing and walking, while peripheral muscles are activated through running and jumping (Armstrong and Laughlin, 1985). Therefore, certain type of exercises can affect different type of muscles in the same limb and elicit different cardio-respiratory responses. In addition, the distribution of type 1 and 2 fibers in and between muscles also depends on type of training and genetics (Simoneau and Bouchard, 1995; Andersen and Aagaard, 2010; Wilson et al., 2012).

Overall, the published experimental results suggest that targeting the muscles with a larger mass and containing a higher proportion of type 2 fibers can be more effective to elicit cardio-respiratory responses. Worth noting again, however, is that type 2 fibers are more susceptible to fatigue (Saltin and Gollnick, 1983).

## Perspectives of Limb Muscles Stimulation as a Therapy to Treat Respiratory Dysfunction Following Spinal Cord Injury

Therapies based on repetitive limb movements and stimulation are becoming more recognized in treating respiratory dysfunctions in people with different medical conditions. For example, upper limb exercising was incorporated in pulmonary rehabilitation for patients with chronic obstructive pulmonary disease (COPD) and demonstrated a reduction in dyspnea episodes (Gigliotti et al., 2005; Ries et al., 2007; Costa et al., 2011; Rekha et al., 2016). In individuals with multiple sclerosis, exercise programs for upper extremities have been shown to significantly improve respiratory function (Mutluay et al., 2007). Strength exercises for upper limbs were reported to be beneficial for respiration in children with cerebral palsy (Shin and Kim, 2017) and stroke patients (Kim and Jang, 2016). Although these two studies were focused on increasing of muscular strength of upper limbs/trunk and normalizing vertebral alignment, the afferent activation of working muscles could also play a significant role in the improvement of respiratory function. As could be predicted from the discussion so far, lower extremity activation can also promote respiratory recovery. Daily electrical stimulation of leg muscles in critically ill patients admitted to the intensive care unit reduced the time for assisted ventilation and patients were weaned earlier that non-stimulated group (Abu-Khaber et al., 2013; Leite et al., 2018). Daily vibratory stimulation over hand and foot proprioceptors has been demonstrated to reduce the number of apneic and hypoxic episodes in premature infants (<34 weeks), supporting again the idea that limb muscle stimulation can facilitate and improve breathing (Kesavan et al., 2016).

Clinical studies also suggest that stimulation of limb muscles may be employed for respiratory rehabilitation in people with spinal cord injuries (SCI). Repetitive activation of the impaired respiratory system by peripheral muscle stimulation might retrain spared neural networks (activity-based therapy) and improve their function post-SCI (Hormigo et al., 2017). This activation can be achieved via supraspinal (in the case of a non-complete SCI) as well as via spinal mechanisms discussed above. Support for this hypothesis can be found in a number of studies that have demonstrated a beneficial effect of lower and upper limb afferent activation on respiratory function in SCI patients. Rhythmic stimulation of lower extremities by assisted treadmill locomotion was able to elicit metabolic responses and increase ventilatory parameters in individuals with chronic complete and non-complete cervical SCI (Nash et al., 2004; Carvalho et al., 2005). Regular treadmill training with body weight support (Terson et al., 2013; Tiftik et al., 2015) or accompanied by functional electrical stimulation (FES) (de Carvalho et al., 2006) significantly increased respiratory motor function in patients with cervical and thoracic SCI. The exercises involving upper extremities (Silva et al., 1998; Le Foll-de et al., 2005) have also been shown to improve ventilatory function in individuals with thoracic SCI.

However, cervical level SCIs are the most devastating and often impair the majority of motor functions below the injury, including paralysis/paresis of both, lower and upper limbs. Treadmill rehabilitation in tetraplegic individual represents a huge logistical and economic burden. Therefore, using FES of upper extremities in people with cervical SCI could be a more suitable alternative. This type of rehabilitation is non-invasive (cutaneous electrode placement), requires less personnel and can be applied in a home environment. Arm-cranking exercise assisted by FES (Hunt et al., 2003; Coupaud et al., 2008) is one of the examples of upper extremities stimulation to enhance respiration in individuals with cervical SCI. Even so, the above studies were not designed to improve respiratory function, a significant increase in oxygen uptake by the end of the training sessions was determined in some participants. Also, it should be noted specifically that combined strategies of FES and exercise (bicycle, arm-cranking, arm-cycling, or their hybrids) show greater potentials for recovery in SCI individuals by producing more effect on cardio-respiratory function (Hettinga and Andrews, 2008; Hasnan et al., 2013). While the clinical studies conducted so far have unfolded exciting results, there is still a great deal that needs to be investigated both clinically and pre-clinically. Reverse translation of these documented studies to animal models of SCI will likely reveal how to optimize these promising strategies for improving ventilation in injured people.

## Conclusion

Overall, despite a significant number of experimental and clinical studies, the detailed mechanisms of respiratory control by stimulation of peripheral muscles in the intact and, especially, injured spinal cord are still unclear and require more thorough investigations. An understanding of these mechanisms will help to develop improved stimulation paradigms and devices for limb FES in combination with different exercises, targeting the most effective choice of muscles to enhance respiratory plasticity and recovery in individuals with cervical SCI and other respiratory dysfunctions. As neural engineering advances the field of neural interfacing, we can expect to see even greater improvements in these described results, with better strategies for improving breathing.

## Author Contributions

NS, VM, and TB made a substantial contribution to the drafting, figure making and discussion of this work and approved it for publication.

## Conflict of Interest Statement

The authors declare that the research was conducted in the absence of any commercial or financial relationships that could be construed as a potential conflict of interest.
